# High prevalence of G3 rotavirus in hospitalized children in Rawalpindi, Pakistan during 2014

**DOI:** 10.1371/journal.pone.0195947

**Published:** 2018-04-30

**Authors:** Massab Umair, Bilal Haider Abbasi, Salmaan Sharif, Muhammad Masroor Alam, Muhammad Suleman Rana, Ghulam Mujtaba, Yasir Arshad, M. Qaiser Fatmi, Sohail Zahoor Zaidi

**Affiliations:** 1 Department of Biotechnology, Quaid-i-Azam University, Islamabad, Pakistan; 2 Department of Virology, National Institute of Health, Chak Shahzad, Islamabad, Pakistan; 3 Department of BioSciences, COMSATS Institute of Information Technology, Chak Shahzad, Islamabad, Pakistan; Instituto Evandro Chagas, BRAZIL

## Abstract

Rotavirus A species (RVA) is the leading cause of severe diarrhea among children in both developed and developing countries. Among different RVA G types, humans are most commonly infected with G1, G2, G3, G4 and G9. During 2003–2004, G3 rotavirus termed as “new variant G3” emerged in Japan that later disseminated to multiple countries across the world. Although G3 rotaviruses are now commonly detected globally, they have been rarely reported from Pakistan. We investigated the genetic diversity of G3 strains responsible RVA gastroenteritis in children hospitalized in Rawalpindi, Pakistan during 2014. G3P[8] (18.3%; n = 24) was detected as the most common genotype causing majority of infections in children less than 06 months. Phylogenetic analysis of Pakistani G3 strains showed high amino acid similarity to “new variant G3” and G3 strains reported from China, Russia, USA, Japan, Belgium and Hungary during 2007–2012. Pakistani G3 strains belonged to lineage 3 within sub-lineage 3d, containing an extra N-linked glycosylation site compared to the G3 strain of RotaTeq^TM^. To our knowledge, this is the first report on the molecular epidemiology of G3 rotavirus strains from Pakistan and calls for immediate response measures to introduce RV vaccine in the routine immunization program of the country on priority.

## Introduction

Rotavirus is the most common etiologic agent of severe diarrhea in infants and children worldwide causing approximately 0.2 million deaths annually [1]. Being a member of *Reoviridae* family, it has a unique genome comprising 11 segments of double stranded RNA which encode six structural (VP1-VP4, VP6, VP7) and six non-structural proteins (NSP1-NSP6) [2]. Among the nine recognized and one proposed rotavirus species (RVA—RVI and RVJ respectively) [3,4] species A, B, C and H are known to infect humans [5] while RVA cause majority of infections [6]. RVA is classified into G and P genotypes based on their outer capsid proteins VP7 and VP4 respectively and 35 G-types and 50 P-types have been identified so far [3,7,8]. Epidemiological studies demonstrated that humans are mostly infected with G1, G2, G3, G4 and G9 in combination with P[4], P[6] and P[8] [9,10] whereas G12 has recently emerged in most parts of the world including Pakistan [11,12].

G3 rotavirus is one of the most common RVA strain reported worldwide [9]. G3 was detected at low rates during 1990’s however during the last decade, it has re-emerged in different countries of the world [13–23]. Moreover, a variant of G3 referred to as "new variant G3" was reported from Japan during 2003–2004 [18]. Later, these variants were also detected from different parts of the world [19,20,23–30]. To date, limited data is available on circulation of RVA genotypes from Pakistan indicating G1, G2 and G9 among the most common genotypes infecting Pakistani children [12,31–34] whereas G3 strains are rarely detected [31]. Therefore, the objective of this study was to investigate the genetic diversity of G3 rotaviruses and to compare them with the two licensed rotavirus vaccines.

## Materials and methods

### Stool specimen collection

Stool samples were collected from children hospitalized with acute dehydrating gastroenteritis at Benazir Bhutto Hospital, Rawalpindi (BBH) as per WHO standard case definition [35] during January to December 2014. Benazir Bhutto Hospital, previously known as Rawalpindi General Hospital is a tertiary care teaching hospital in district Rawalpindi with its pediatric department providing health facilities to population of Rawalpindi (the fourth largest city by population in the country) and its adjacent regions. The inclusion criteria of enrolled cases followed was: any child under five years of age admitted to the hospital for the treatment of acute gastroenteritis as primary illness and onset of symptoms in ≤ 7 days. The cases aged above 5 years and those presented with bloody diarrhea and non-infectious gastric disorders were excluded from the study. Informed written consent was obtained from patient’s parent/guardian before sample collection. Demographic and clinical data including age, gender, area of residence, hospital admission date, date of stool sample collection, vomiting (duration and episodes per 24 hours), diarrhea (duration and episodes per 24 hours), body temperature and patient’s recovery status at the time of discharge from hospital was recorded on a standard questionnaire. Stool samples and the relevant clinical records of patients were transported to Department of Virology, National Institute of Health (NIH), Islamabad for laboratory testing. The study design was approved by Internal review Board of the National Institute of Health, Islamabad.

### Enzyme immunoassay

The presence of Group A rotavirus in stool was confirmed by use of a commercial immunoassay (ProSpecT, Oxoid Ltd.) following manufacturer instructions.

### Viral RNA extraction and G & P genotyping

RNA was extracted from 10% fecal suspension using QIAamp Viral RNA Mini Kit (Qiagen Hilden, Germany) according to manufacturer’s instructions and stored at -20°C until further analysis. Reverse-transcription polymerase chain reaction (RT-PCR) assay and G and P typing was carried out as described previously [36,37].

### Sequence analysis of VP7 and VP4 genes

Partial sequencing of VP7 and VP4 genes was directly done with primers Beg9 & End9 [36] and Con2 & Con3 [37] respectively using BigDye Terminator Cycle Sequencing kit v3.0 (Applied Biosystems) on an automated sequencer ABI 3100 (Applied Biosystems). Analysis of nucleotide sequences was done by using Sequencher version 4.1 (Gene Codes Corp., USA). Closely matched nucleotide sequences of VP7 and VP4 genes were retrieved from GenBank using BLAST (https://blast.ncbi.nlm.nih.gov/Blast.cgi). Phylogenetic tree was constructed using neighbor joining method (with 1000 bootstrap replicates) and distances were calculated with Kimura 2-parameter method using MEGA 5 [38]. Lineage designation for phylogenetic trees of G3 and P[8] were based on criteria reported earlier [19,39]. Structural analysis of VP7 (PBD 3FMG) and VP8* (PDB 1KQR) were performed using the UCSF Chimera-Molecular Modeling System [40]. Nucleotide sequences of rotavirus VP4 and VP7 gene segments sequenced in this study are submitted to the GenBank under the accession numbers KX681820-KX681833.

## Results

### Clinical features of RVA G3 patients

From a total of 502 patients, G3P[8] (18.3%; n = 24/131) was detected as the most common genotype. In the current study the highest rate of infection due to rotavirus G3 was found in children aged 1–6 months (56.6%, 17 out of 30) followed by those between 7–12 months of age (40%, 12 out of 30). Infection with G3 rotavirus was observed in a single child of 24 months age. Frequency of G3 infection in male was higher (63.3%) than female (36.7%) patients. Infections with G3 were found throughout the year and the most common clinical signs and symptoms of children infected with G3 included dehydration (100%), vomiting (mean duration = 2.3±4.1 days, range = 1–6 days and mean episodes per 24 hours = 3.6±6.21, range = 1–10 days) and diarrhea (mean duration = 2.3±3.1 days, range = 1–6 days and mean episodes per 24 hours = 12.5±15.3, range = 10–16 days).

### Phylogenetic analysis

**Fig 1:**
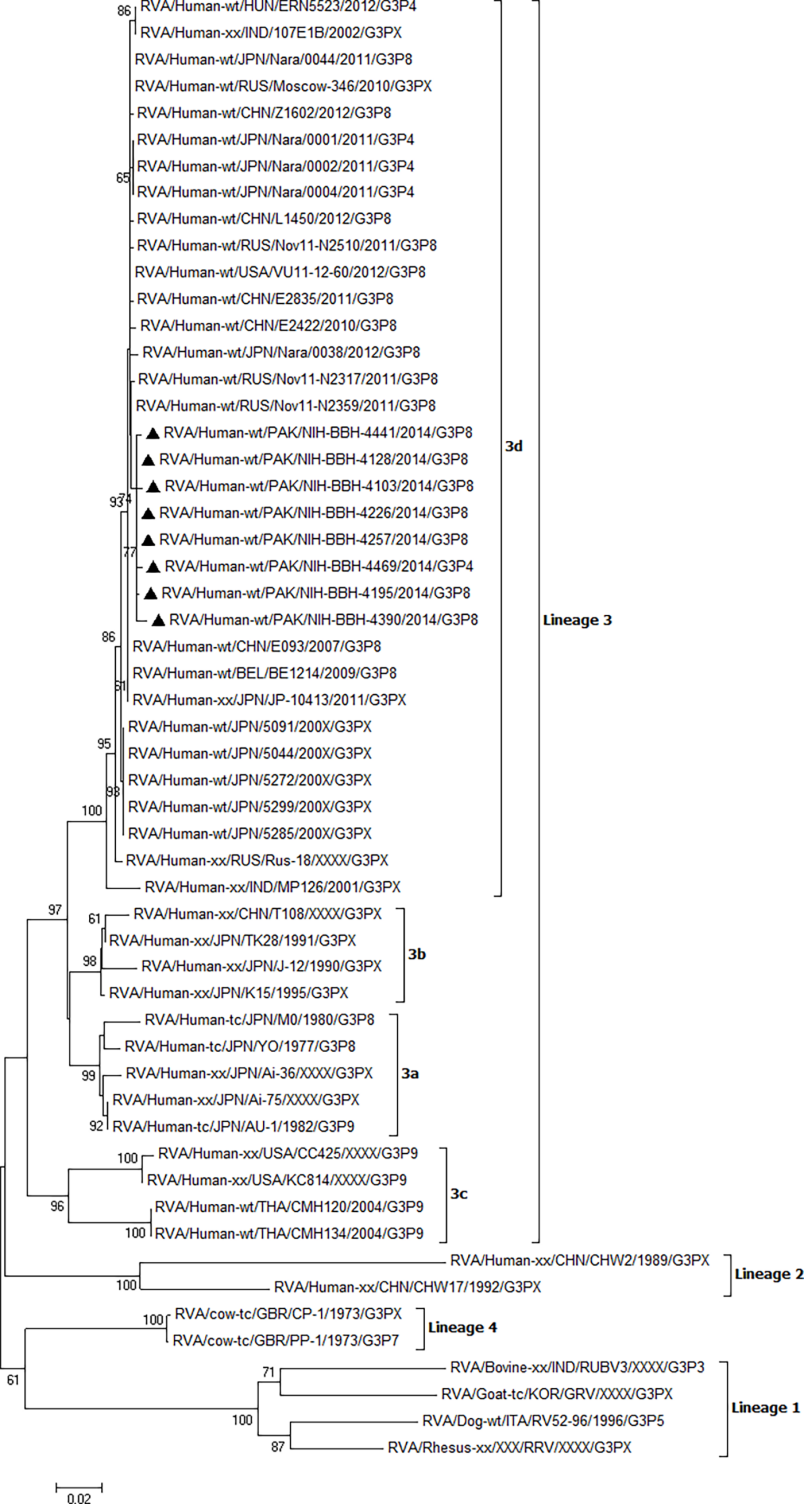
Phylogenetic analysis of VP7 gene segment of G3 rotavirus strains. Phylogenetic tree was reconstructed using neighbor-joining method. Bootstrap values were calculated using 1000 replicates. Bootstrap values less than 60 are not shown. Filled triangles represent G3 strains detected in this study.

In order to explore the genetic diversity of G3, eight strains were randomly selected and their VP7 gene was partially sequenced (942bp). Phylogenetic analysis showed that all Pakistani G3 strains shared high nucleotide and amino acid similarity (99–100%) with each other and 98.9–99.8% similarity was observed between Pakistani and recent G3 strains reported from China, Russia, USA, Japan, Belgium and Hungary within lineage 3 and sub-lineage 3d (Fig 1). These strains showed 98.7–99.1% similarity at nucleotide and 99–99.7% at amino acid level respectively with new variant G3 isolated in Japan during 2003–2004 [18]. Deduced amino acid sequence of VP7 gene between Pakistani and new variant G3 was highly conserved except for position 221 with Adenine substituted to Aspartic acid (A to D).

**Fig 2:**
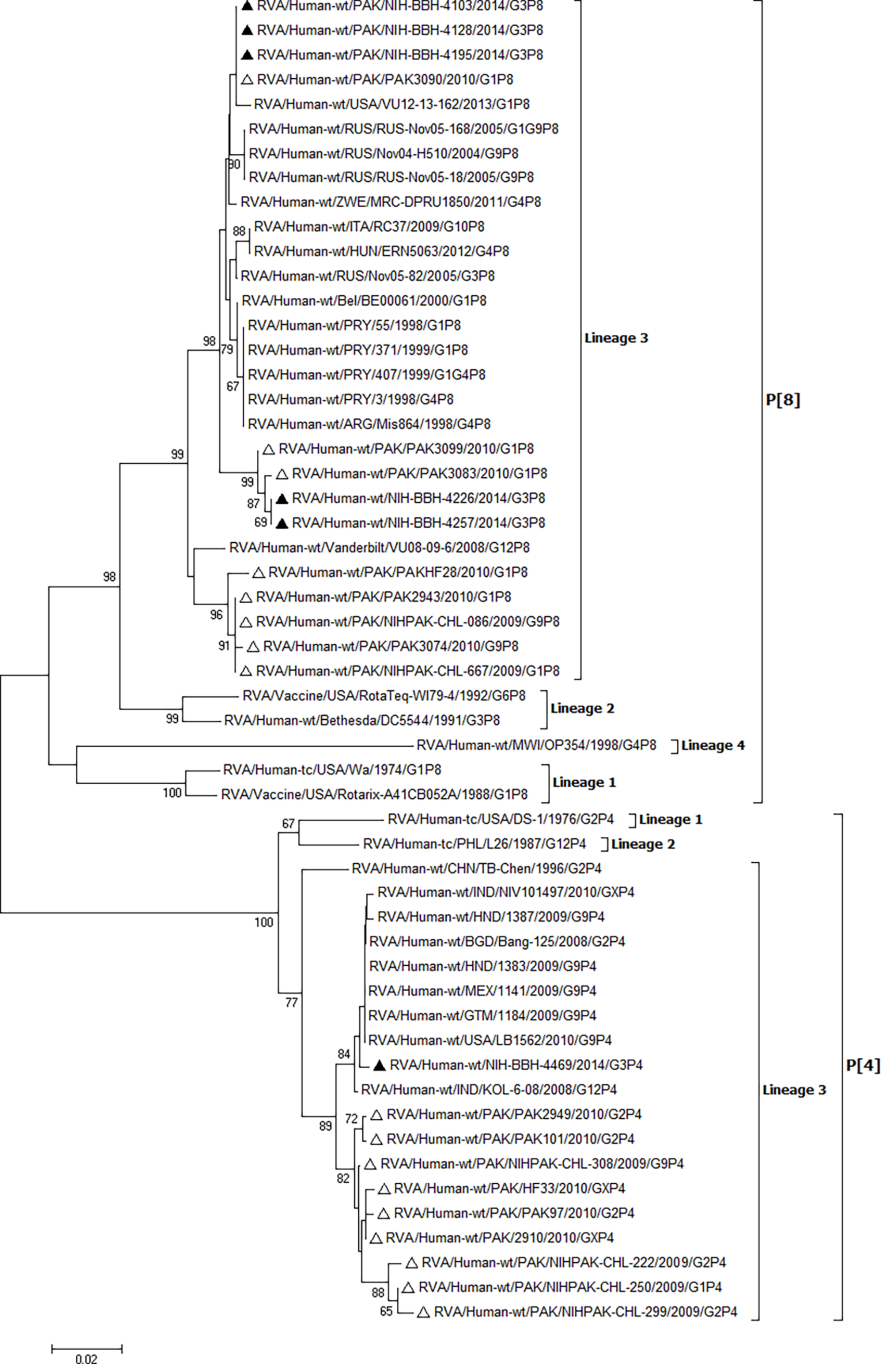
Phylogenetic analysis of rotavirus VP4 gene segment. Phylogenetic tree was reconstructed using neighbor-joining method. Bootstrap values were calculated using 1000 replicates. Bootstrap values less than 60 are not shown. Filled triangle represent P[8] and P[4] strains detected in this study. Empty triangle represent P[8] and P[4] strains reported previously from Pakistan.

Partial sequencing of VP4 gene of five G3P[8] and one G3P[4] strain was also carried out. All five Pakistani G3P[8] strains shared high identities (98.2–100% at nucleotide and 98.9–100% at amino acid level) with each other and were closely related to P[8] strains isolated from USA, Russia, Belgium, Italy, Argentina, Hungary, Zimbabwe and Paraguay (Fig 2). The Pakistani G3P[4] clustered in lineage 3 with P[4] strains from USA, Mexico, Honduras, Guatemala, Bangladesh and India (Fig 2).

### Comparison of Pakistani G3 strains with RVA vaccine strains

**Fig 3:**
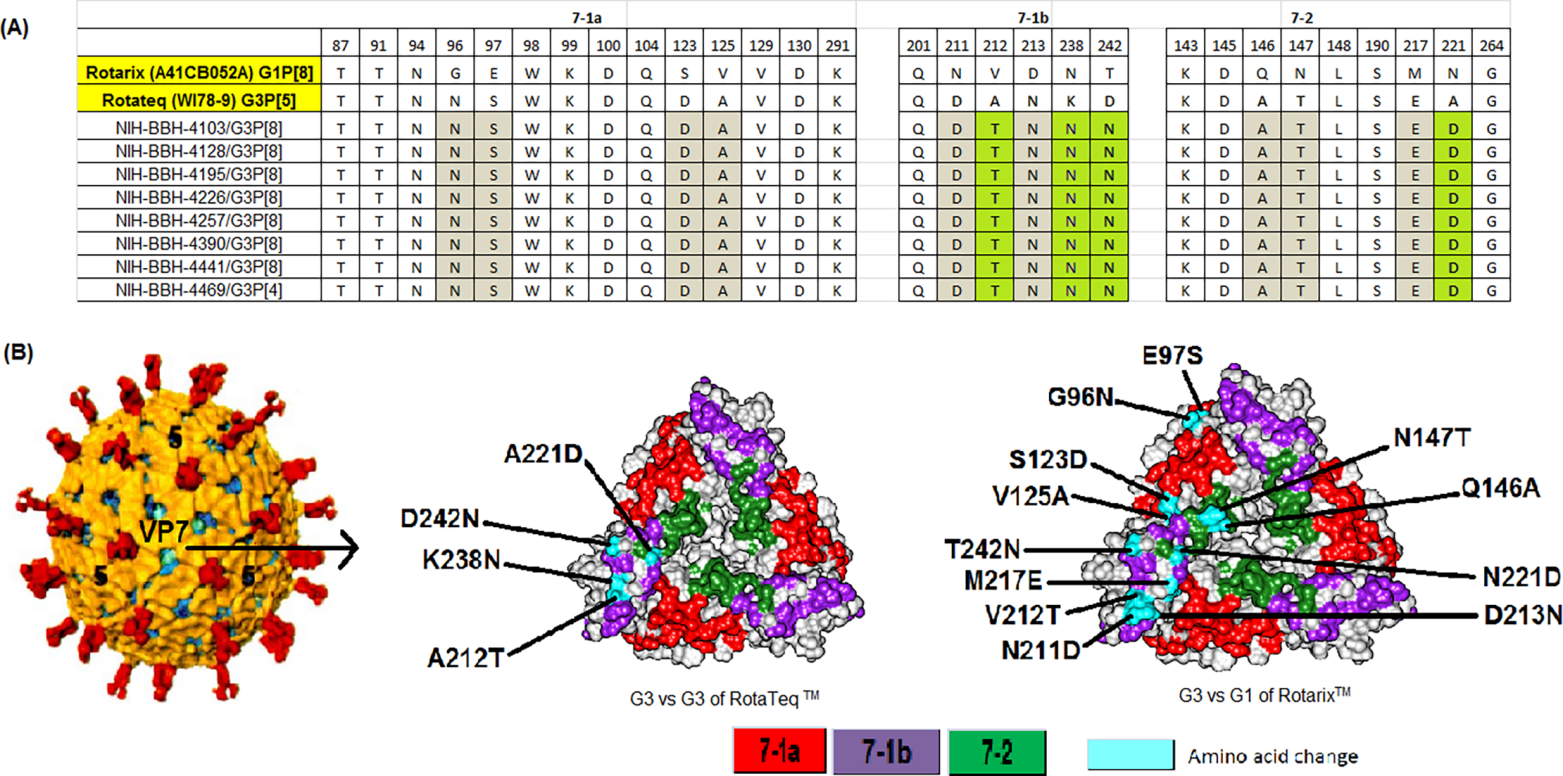
Comparison of VP7 antigenic epitope sites between Pakistani G3 strains and rotavirus vaccine Rotarix^TM^ and RotaTeq^TM^. **(A)** Antigenic residues are divided into three antigenic epitopes 7-1a, 7-1b and 7–2. Amino acid highlighted in green are those that differ from G3 strain of RotaTeq^TM^ while those in gray are different from Rotarix^TM^. **(B)** Surface representation of VP7 trimer (PDB 3FMG). Antigenic epitopes are colored in red 7-1a, purple 7-1b and green 7–2. Surface exposed residues that differ between Pakistani G3 and vaccine strains of Rotarix^TM^ and RotaTeq^TM^ are shown in cyan.

In the present study, we compared the VP7 and VP4 (VP8*) antigenic epitopes of Pakistani G3 with those of RVA vaccine strains. Of the 29 amino acid residues comprising VP7 antigenic epitopes, four differences were found between RotaTeq^TM^ (WI78-9) and Pakistani G3 strains, three of which occurred in 7-1b region (A212T, K238N, D242N) and one difference was found in the antigenic epitope 7–2 (A221D) (Fig 3). Interestingly, all Pakistani G3 strains showed a K238N substitution, which created a potential N-linked glycosylation site in contrast to G3 strain of RotaTeq^TM^. When compared with Rotarix^TM^, twelve amino acid changes were observed within the VP7 antigenic epitopes that were equally distributed among 7-1a, 7-1b and 7–2 (Fig 3).

**Fig 4:**
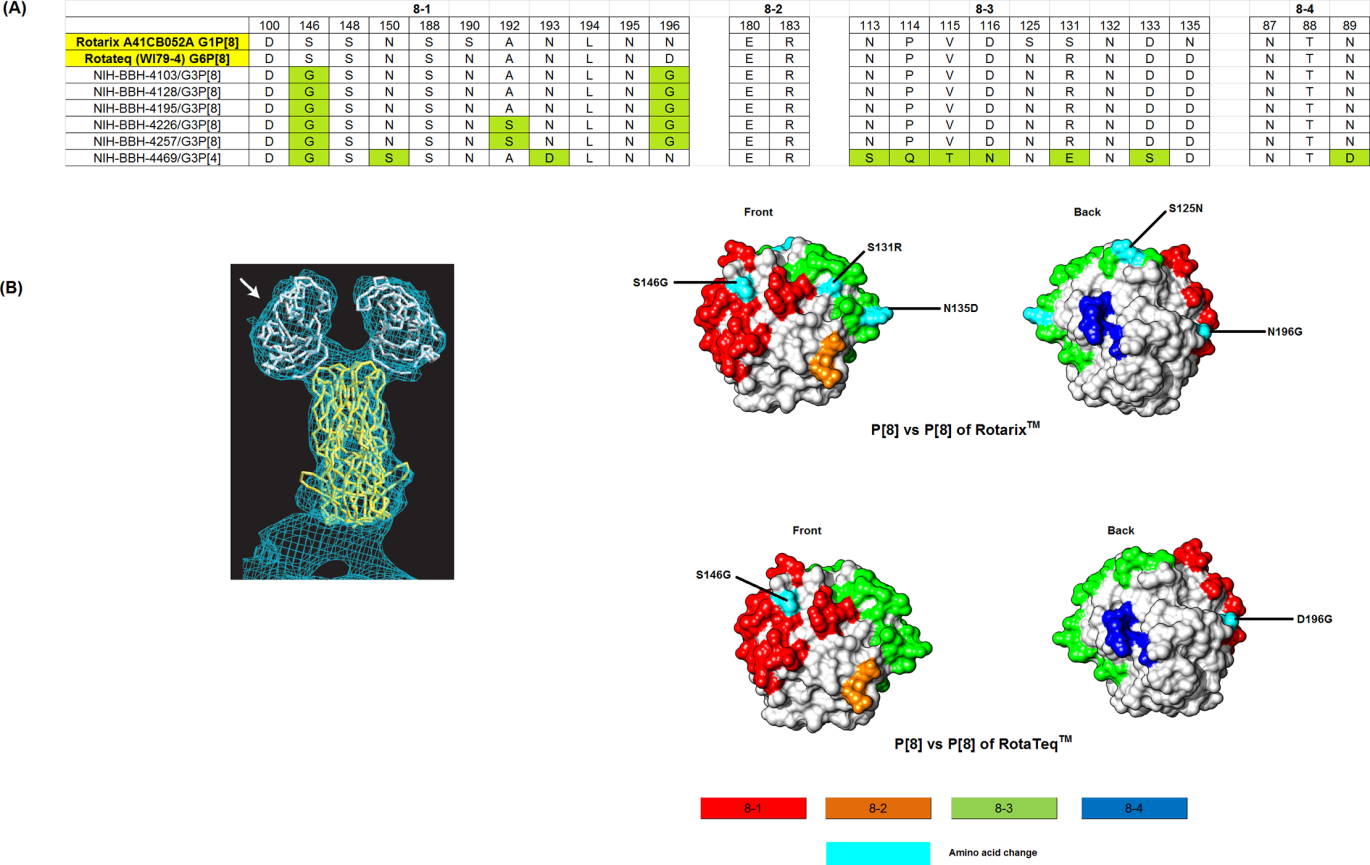
Alignment of antigenic residues in VP4 between the strains contained in Rotarix^TM^ and RotaTeq^TM^ and Pakistani P[8] and P[4]. **(A)** Antigenic residues are divided in three antigenic epitopes in VP8* (8–1, 8–2, 8–3 and 8–4). Amino acids in green are different from both Rotarix^TM^ and G3 strain of RotaTeq^TM^. **(B)** Surface representation of the VP8* core (PDB 1KQR). Antigenic epitopes are colored red 8–1, orange 8–2, green 8–3 and blue 8–4. Surface exposed residues that differ between Pakistani G3P[8] and vaccine Rotarix^TM^ and RotaTeq^TM^ are shown in cyan.

VP8* antigenic epitopes (8–1, 8–2, 8–3, 8–4) of Rotarix^TM^ and RotaTeq^TM^ were compared with those observed in P[8] and P[4] strains detected in this study. Out of total 25 amino acids in the VP8*, ten were found conserved among vaccine and Pakistani strains (Fig 4A). Pakistani G3P[8] strains showed 6 amino acid differences when compared with Rotarix^TM^ in 8–1 and 8–3 epitopes, while two variations were found when compared with RotaTeq^TM^ (Fig 4). Thirteen amino acid differences were observed between Pakistani G3P[4] (strain NIH-BBH-4469) and Rotarix^TM^ while 11 variations were observed when compared to RotaTeq^TM^ (Fig 4A).

## Discussion

Studies on clinical epidemiology of G3 rotavirus strains reported globally are very scarce. In a 2009 study conducted by Day *et al* [41] in Bangladesh, 2.6% of the total 917 enrolled patients were detected positive for G3 rotavirus. In their report, the majority (78%) of G3 positive cases was below 12 months of age and all patients showed dehydration. Vomiting was reported in 55% patients with 5–8 diarrheal episodes in a day. In our study, 100% of the patients were presented with vomiting, dehydration and watery diarrhea. The mean diarrheal episodes in our patients were remarkably high with mean number of 12.5±15.3 episodes per 24 hours. In another study conducted in Japan, Phan *et al* [18] tested 402 fecal specimen from patients with acute gastroenteritis reported during 2003–2004, out of which 21% (n = 83/402) were found positive for rotavirus. Genotyping results indicated that 81 out of 83 positive patients were infected with G3 rotavirus with the highest positivity rate among children above 12 months of age. This is in contrast to our findings where we found 56% infants positive for rotavirus below 6 months of age.

RVA G3 is a ubiquitous genotype but it was only reported from Faisalabad region of Pakistan in 2010 at very low levels [31]. Notably, our results highlight a greater degree of G3 detection compared to previous studies in the region [31]. The high detection rate (22.9%) of G3 in our study is consistent with reports from Japan [18,19,24], China [20,42], Vietnam [43] and Hong Kong [25].

G3 rotaviruses detected in Japan during 2003–2004 contained multiple amino acid changes in the VP7 gene consequently named as “new variant G3” represented by 5091 strain [18]. These viruses continued to circulate in Japan from 2007 to 2011 [19,24]. Pakistani G3 strains detected in this study were highly identical to the new variant G3 found in Japan as well as to the contemporary G3 strains circulating worldwide [44–46]. Similarly, high degree of amino acid similarities (99–99.7%) were observed between Pakistani G3 and Chinese new variant G3 isolated during 2010–2012 [46]. China has a long border with Pakistan and there is frequent movement of people across the border for trade reflecting a possible source of introduction from China into Pakistan or vice versa.

Comparison of antigenic epitopes of RVA vaccine RotaTeq^TM^ and Pakistani strains revealed that all Pakistani G3 strains contained K238N change in VP7 gene which creates a potential N-linked glycosylation site. The presence of this glycosylation site might change the antigenicity of G3 strains and calls for further investigations. Previous reports have shown that glycosylation of 238 residue can reduce neutralization of animal RVA strains by hyper-immune sera and monoclonal antibodies [47,48]. Furthermore, the role of glycosylation of viral proteins in altering the immunogenicity of different viruses like influenza A virus, human immunodeficiency virus, and human respiratory syncytial virus has previously been reported [49–51].

Pakistan has an annual birth rate of approximately 5 million and is among the five countries where rotavirus related deaths are the highest [52] hence a suitable rotavirus vaccine is the need of time. Two vaccines (monovalent Rotarix^TM^ and pentavalent RotaTeq^TM^) are currently licensed for immunization against rotavirus worldwide [53]. WHO recommends the inclusion of these vaccines into national immunization programs of all countries especially those where diarrheal deaths account for ≥10% of under-five mortality [54]. The effectiveness of these vaccines is higher in developed countries compared to low and middle income countries [55–59]. Pakistan is expected to introduce RVA vaccine in its Expanded Program on Immunization (EPI) in 2017 with the support of GAVI (Global Alliance for Vaccines and Immunization). The success of the rotavirus vaccines in Pakistan will depend on their ability to provide protection against the RVA genotypes circulating in the country. Considering the variations observed in the VP7 and VP4 epitopes of Pakistani G3 strains and those of vaccine strains, further investigation of the antigenic variability and their impact would be required to understand the significance of these differences and their influence on vaccine efficacy.

To our knowledge, this is the first report on the molecular epidemiology of G3 and detection of new variant G3 rotavirus from Pakistan. We could not compare our G3 strains with those previously reported from Faisalabad, Pakistan [31] due to the unavailability of VP7 sequences in the GenBank. In addition, the small sample size was a limitation of the current study. However, our present findings conclude that rotavirus G3P[8] genotype is a major cause of acute gastroenteritis in pediatric population admitted in a tertiary care hospital of Rawalpindi. Comparative analysis of Pakistani G3 with RVA vaccine Rotateq^TM^ showed a K238N change in the VP7 gene which creates a potential N-linked glycosylation site. The genotypic diversity and high prevalence of G3 strains detected in the current study highlights the need for robust large scale surveillance to formulate future vaccine strategies.
